# Normative Database of Retinal Oximetry in Asian Indian Eyes

**DOI:** 10.1371/journal.pone.0126179

**Published:** 2015-04-29

**Authors:** Ashwin Mohan, Supriya Dabir, Naresh Kumar Yadav, Matthew Kummelil, Rajesh S. Kumar, Rohit Shetty

**Affiliations:** Narayana Nethralaya, Post Graduate Institute of Ophthalmology, Bangalore, India; University of Melbourne, AUSTRALIA

## Abstract

**Objective:**

To study the oxygen saturation profile in normal Asian Indian eyes.

**Design:**

A cross sectional prospective study.

**Subjects:**

Ninety eight consecutive patients presenting to our hospital with best corrected distance visual acuity (BCVA) of 20/20 and a normal ophthalmic examination were included in the study. Patients having any ocular or systemic disease were excluded from the study.

**Materials and Methods:**

Oximetry was performed on all subjects with the Oxymap T1 retinal oximeter (Oxymap hf, Reykjavik, Iceland).

**Main Outcome Measures:**

The images were analysed for oxygen saturation and diameter.

**Results:**

The mean age was 33 years (Range: 18-63; SD: 12.4). The average arteriolar saturation was 90.3 ± 6.6% and the venous saturation was 56.9% ± 6.3. The average A-V (arterio-venous) difference was 33.2% ± 5.2. There was an increase in arteriolar (R^2^ = 0.264; p=0.001) and venous saturation (R^2^ = 0.151; p=0.001) with age. There was no significant change in the arterio-venous saturation difference (AVSD). The inferotemporal quadrant had the lowest saturations. Age correlated positively with ocular perfusion pressure (OPP)(R^2^ = 0.07; p=0.007). OPP correlated positively with global arteriolar saturation (R^2^=0.057, p=0.018).

**Conclusion:**

This study provides the normative database for an Indian population and is comparable to previous studies. Age, vessel diameter and OPP were the significant factors that influenced the saturation. Arteriolar and venous saturations increased with age while the AVSD did not change significantly.

## Introduction

Oxygen is important for the normal physiology and metabolism in the body. The retina has the highest metabolic demands of any tissue in the body with the photoreceptors having the highest demand in the retina.[1] Inadequate delivery of oxygen or altered utilization of oxygen can either trigger disease or be a marker for underlying disease activity respectively.[2–5]

Hemoglobin bound to oxygen has differential absorption of light at different wavelengths. This is the basic principle used in dual wavelength oximetry. Fundus photographs at 2 different wavelengths are taken and the relative oxygen concentration can be determined using photospectrometry. [6,7] Jani et al[8] in their work state that this machine is based on a similar principle as the finger pulse oximeter.

Previous studies[8,9] have established a normative database using the Oxymap T1 Retinal Oxymeter (Oxymap, Reykjavik, Iceland). We have used the same equipment in our study. Jani et al[8] showed in their multiethnic study that race and pigmentation when uniform do not play a role. We wanted to evaluate this in an Indian population.

It has been shown that narrower retinal vessels show higher oxygen saturations[9]. In contrast, in another study[8] the diameter had no impact on the measured saturation as the software compensated for the optical artifacts caused by the diameter. In view of the conflicting results, we intend to establish our own results.

We aim to study averages and variability of oxygen saturation in healthy individuals in an Indian population. We also aim to study the interocular variability, quadrant values, relationship with diameter and age.

## Methods

Healthy subjects presenting to our outpatient department at Narayana Nethralaya, Bangalore were enrolled in the study with their consent. The study follows the tenets of Helsinki. The Institutional Review Board and Ethics Committee approved the study.

### Inclusion

Enrolled subjects had a normal ophthalmic examination, BCVA 20/20–20/30, with spherical refractive error less than ±6.00 diopters and cylindrical error of less than ±2.00 diopters.

### Exclusion

Patients with BCVA vision less than 20/30, cataract greater than Lens Opacification Classification System—Nuclear sclerosis Grade 2/Posterior Subcapsular Cataracts (NS2/PSC) or other significant media opacities, history of ocular conditions such as diabetic or hypertensive retinopathy, macular degeneration, or retinal vascular occlusions or chronic systemic conditions like diabetes or hypertension that can confound retinal oximetry measurements were excluded. Patients regularly taking any systemic drugs or smokers were also excluded.

### Image Acquisition

All patients underwent dilation with 1% tropicamide and 10% phenylephrine and subsequent retinal imaging with the oximetry using the Oxymap T1 Retinal Oximeter (Oxymap, Reykjavik, Iceland). The procedure was explained to them in detail and a written informed consent was obtained. They were allowed to rest for 5 minutes before the images were captured. This was done to eliminate exercise induced fluctuations in readings. Resting blood pressure and pulse oximetry (H5, Silicon Labs, India) readings were taken for all patients. None of the subjects had consumed caffeine within 2 hours of the examination.

The aiming light was set at the lowest setting, flash intensity was 50W, small aperture and large pupil settings were applied to the TRC 50DX (Topcon, Japan) Fundus camera.

**Fig 1:**
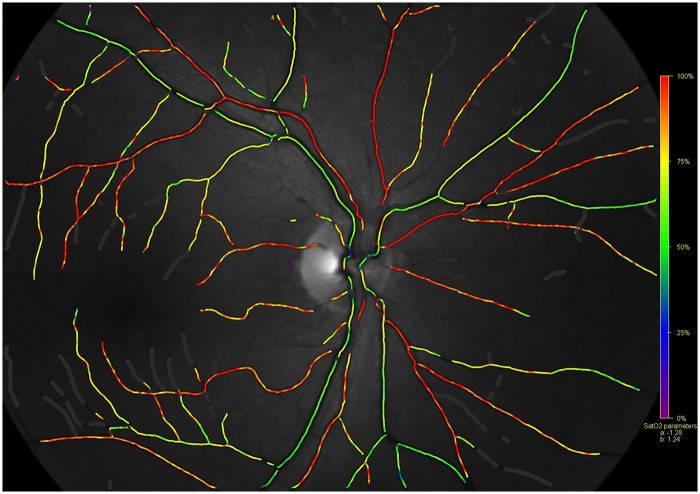
A normal Oxymap image with the pseudo-colour saturation overlap map (Background image—570 nm).

One experienced photographer obtained standardized images for all the subjects. We obtained 2 images per eye in all the subjects—50°—disk centered (Fig 1) and macula centered. Defocus can cause decrease in contrast and hence alterations in the measured values. We ensured that all the images analysed were in sharp focus.

### Segment selection and Analysis

**Fig 2:**
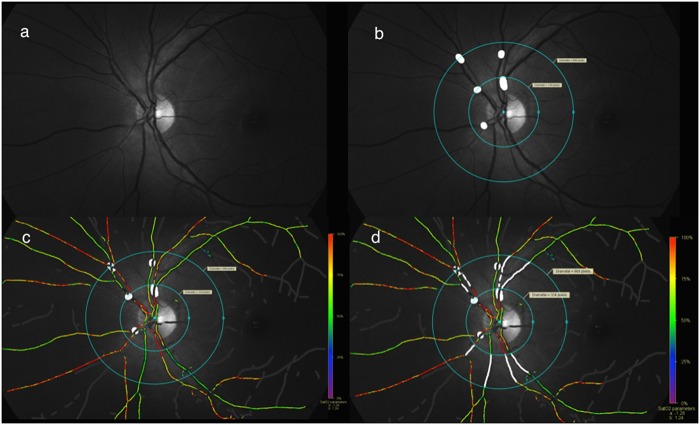
Method of analyzing images. a. 570nm image; b. Masking of vessel branchings and A-V crossings along with marking of 2 concentric circles within which vessel segments would be analysed; c. Pseudo-color map along with previous markings; d. Vessel segment markings (thickest arteriole and venule per quadrant)

The software used was version 2.4.2. The oxygen saturation was given by the equation: saturation = (a x ODR + b)100; a = -1.28 and b = 1.24. Segments of vessels (Fig 2) were then chosen according to the protocol described by Geirsdottir et al[9]. The vessels were atleast 8 pixels wide (72 μm), atleast 100 pixels long, were minimum 50 pixels from the optic disc margin and at least 100 pixels from the edge of the image. In cases of vessel branchings which were less than 150 pixels from the optic disc, segments after the branching were taken. All images had uniform background pigmentation. Arteriolar and venous saturations were determined separately for superior, inferior, nasal and temporal quadrants. For analyzing the quadrant values, we selected the thickest arteriole and venule in each quadrant without any weighted averaging. Global values were also determined by averaging the quadrant values.

### Statistical Analysis

Statistical analysis was done using the IBM SPSS v22 software. All continuous variables were tested for normality using the Shapiro-Wilk test. The difference between means was tested using independent t-test for normally distributed data and the Mann-Whitney U test for non-parametric data. For normally distributed data the Pearson’s correlation was used, for non parametric data the Spearman’s correlation was used. The level of significance was p<0.05. Linear regression was performed after identifying significant correlations.

## Results

**Fig 3:**
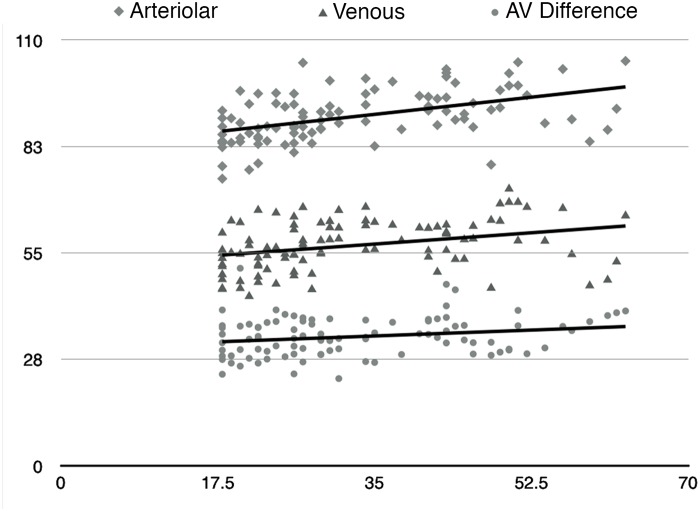
Relationship of Arteriolar, Venous and A-V Difference Saturations with age. (x-axis—Age [Years]; y-axis—Saturation [%])

There were a total of 98 eyes (98 subjects). The mean age was 33 years (18–63)(SD = 12.4) (Fig 3). There were 44 males and 54 females. No statistically significant bilateral differences were noted and hence only the right eyes were used for the statistical data, hence avoiding duplication. The arteriolar and venous saturations were normally distributed (Shapiro-Wilk Test). The average arteriolar saturation was 90.3 (95% C.I 88.9–91.6) (Standard deviation (SD): 6.57, skewness: 0.086, kurtosis: -0.257) and the venous saturation was 56.9 (95% C.I 55.7–58.2) (SD: 6.31, skewness: -0.004, kurtosis: -0.678). The average A-V (arterio-venous) difference was 33.2 (95% C.I—32.2–34.3)) (SD: 5.18, skewness: 0.369, kurtosis: 0.864). The other parameters like diameter and quadrant wise saturation are summarized in Table 1.

**Table 1:** Summary of the Global and Quadrant saturations and diameters.

	Global			Quadrant—Arteriolar				Quadrant—Venous			
	Arteriolar	Venous	AV Difference	ST	SN	IN	IT	ST	SN	IN	IT
**Saturation (%)**	90.3 ± 6.6	56.9 ± 6.3	33.2 ± 5.2	88.0 ± 7.9	93.9 ± 8.8	93.0 ± 9.2	86.2 ± 8.6	56.0 ± 6.7	60.0 ± 7.6	60.2 ± 8.8	51.4 ± 6.8
**Diameter (μm)**	123.4 ± 11.2	159.7 ± 12.7		134.3 ± 16.8	111.3 ± 16.8	111.1 ± 15.6	136.4 ± 17.7	171.4 ± 21.7	142.9 ± 19.8	141.7 ± 21.2	182.2 ± 20.6

AV difference—arterio-venous difference; ST—supero-temporal; SN- supero-nasal; IN—infero-nasal; IT—infero-temporal.

### Effect of age

There was an increase in arteriolar (R^2^ = 0.263, p = 0.001) and venous saturation (R^2^ = 0.151, p = 0.001) with age. The A-V difference did not show a significant change with age. The trends have been summarized in Fig 3. The average ocular perfusion pressure (OPP) was 43.6 (95% C.I 42.1–45.1)) (SD: 7.3, skewness: 0.019, kurtosis: -0.526). This showed a positive correlation with age (R^2^ = 0.073; p = 0.007) and with the global arteriolar saturations (R^2^ = 0.057, p = 0.018)

### Temporal vs Nasal analysis

The average arteriolar saturation (%) was 87.1 (95% C.I 85.9–88.3) temporally and 93.5 (95% C.I 92.2–94.8) nasally (p = 0.001); whereas the venous saturation was 53.7 (95% C.I 52.7–54.7) temporally and 60.1 (95% C.I 59.0–61.3) nasally (p = 0.001). The average arterial and venous diameters (μm) were 135 and 177 temporally; whereas they were 111 and 142 nasally (p = 0.001,p = 0.001). Both the temporal (R^2^ = 0.04, p = 0.007) and nasal (R^2^ = 0.03, p = 0.026) saturations correlated positively with the OPP. The inferotemporal quadrant had the lowest saturations with 86% and 51%, highest diameters with 136μm and 182μm, arteriolar and venous respectively.

### Eye Laterality

There were no statistically significant bilateral differences noted in either the quadrants or the global arteriolar, venous or A-V difference saturations.

### Multivariate regression

A multiple linear regression was performed for the dependent variables namely the arteriolar, venous and A-V difference saturations by taking the independent variables as age, sex, ocular perfusion pressure and spherical equivalent. The results have been summarized in Table 2. The naïve analysis of all the variables in the table yielded a significant correlation; age was the only significant variable in the multivariate model for global arteriolar and venous values.

**Table 2:** Multivariate analysis for arteriolar, venous and A-V difference saturations (only those parameters significant on univariate analysis were included in the multivariate analysis).

Variable	Arteriolar		Venous		A-V Difference	
	Standardised Co-efficient	P Value	Standardised Co-efficient	P Value	Standardised Co-efficient	P Value
Intercept	76.64	<0.001	50.90	<0.001	33.66	<0.001
Age	0.41	<0.001	0.263	0.028	-0.077	0.542
Spherical Eq	0.066	0.507	0.064	0.548	-0.007	0.950
Systolic	0.039	0.810	0.088	0.614	-0.077	0.678
Diastolic	0.048	0.821	-0.232	0.314	0.017	0.944
OPP	0.053	0.832	0.220	0.412	0.108	0.706

### Effect of diameter

We found a negative correlation between diameter and saturation for individual vascular segments (arterioles: R^2^ = 0.06, p = 0.001; venules: R^2^ = 0.05, p = 0.001). Globally average diameter and saturation showed a weak negative correlation which was not statistically significant.

## Discussion

Our results on normative Asian Indian eyes using the Oxymap T1 retinal oximeter yields an average saturation of 90.3% in the arterioles and 56.9% in the venules which is similar to previous studies [8,9] which give an average arteriolar value of 90.4–92.2% and a venous value of 55.3–55.6%.

### Pulse Oximetry and the eye

The difference between the average pulse oximetry readings and the average arterial SO_2_ is 7%, but no statistical correlation was found between the two parameters. The standard deviation of the pulse oximetry readings is only 2 as compared to 7 with oximetry.

### Perfusion pressure

Geirsdottir et al have in their study showed the ocular perfusion pressure increases with age.[9] We found a similar trend in our study as well. This would cause the blood to course faster through the retinal vasculature.

### Effect of aging

Tissue loss with aging[10] should result in lower oxygen extraction and hence higher saturations. There is also a decrease in diameter with age. This too can result in optical artifacts, artificially raising the saturations. Aging can also result in subtle changes in the lens resulting in decreased contrast in the acquired images. This can cause a lower measured saturation.[11] We found an increase in arteriolar, and venous saturations with age, leaving the A-V difference unaffected. We feel that decreased diameter, decreased retinal nerve fiber layer thickness and increased perfusion pressure would lead to this trend. We attribute the increased saturation in arterioles and venules found in our study to multiple factors. An opposite trend was noted in the previously mentioned studies. Retinal tissue is lost with aging, hence there are 2 important events that occur. The first being that the oxygen requirement and extraction decreases hence increasing saturation. In the setting of decreased tissue A-V difference should decrease. But here we are looking at oxygen extraction which is a product of A-V difference x blood flow. Blood flow is heavily dependent on diameter which decreases with age, as a result of age related arteriolosclerosis. This means the total amount of blood flowing through the human retina decreases as age advances. Hence a decrease in extraction with an associated decrease in blood flow may explain the unaffected A-V difference with age. The second important event occurring with advancing age is a theoretical decrease in the reflectivity due to loss of RNFL. This might not manifest significantly in Caucasians, but in more heavily pigmented fundi like in Indians, more melanin is exposed, hence absorbing more light. This could alter the final measured Optical Density Ratio (ODR), hence altering saturation measurements. These are hypotheses that need to be tested. There is also a possibility that the Retinal Nerve Fiber Layer (RNFL) responds differently at 570nm and at 600nm. Jani et al state that fundus pigmentation did not affect measurements, but maybe all those subjects had a good RNFL thickness not allowing the complete role of melanin to manifest.

The vessel walls change with age owing to sclerosis, which may alter the amount of light that interacts with the blood column and thus prevents us from measuring the true saturation. Our patients were methodically screened and we ensured that none of them had systemic hypertension or diabetes. It would be interesting to see how saturations vary with varying grades of hypertensive retinopathy. On one hand increased perfusion pressure and blood flow velocity may alter saturation, and altered vessel wall characteristics may change saturation on the other.

### Conclusion

This study provides the normative database for an Indian population and is comparable to previous studies. Age was the most significant factor that influenced the saturation. We also see that diameter and saturation of individual vessel segments are inversely correlated. Arteriolar and venous saturations increased with age whereas A-V difference saturations remained unchanged in this study. It is essential to understand the normal variations in the saturation to be able to pick up subtle changes in pathology.
